# Freedom, conflict, and the eclipse of emancipation: a positional sociology perspective

**DOI:** 10.3389/fsoc.2026.1783568

**Published:** 2026-02-23

**Authors:** Fabio de Nardis

**Affiliations:** Centre for Conflict and Participation Studies, Department of Human and Social Sciences, University of Salento, Lecce, Italy

**Keywords:** conflict, emancipation, freedom, positional sociology, social inequality

## Abstract

This article offers a critical sociological analysis of the contemporary disjunction between the persistent emphasis on individual freedom and the progressive weakening of the material and collective conditions of emancipation. Rather than interpreting this disjunction as a simple gap between normative principles and social practices, the article argues that it reflects a structural transformation in the meaning of freedom and in its relationship to conflict. The analysis shows how freedom, in its hegemonic contemporary configuration, has historically consolidated as a status. Understood as a formal attribute of the subject, freedom is not restricted but stabilized in a way that separates it from the social relations and material conditions that enable its effective exercise. This reconfiguration allows freedom to coexist with growing inequalities, widespread precarity, and institutionalized forms of violence, without requiring any transformation of the social relations that produce them. From this perspective, emancipation does not appear as the unfulfilled complement of freedom, but as what freedom, as status, tends structurally to displace. Conceived as a historical and conflictual process, emancipation makes visible the conditions of subjectivation and challenges the apparent necessity of the existing social order. Its marginalization therefore signals not its theoretical obsolescence, but the increasing difficulty of sustaining collective processes of social transformation in contexts marked by the depoliticization of conflict. Drawing on a positional sociology perspective, the article conceptualizes freedom and emancipation as situated and unequally distributed capacities, and proposes a framework for reconnecting freedom, conflict, and emancipation within contemporary critical social theory.

## 1 Freedom, conflict, emancipation

In the history of political modernity, freedom and conflict constitute a structurally ambiguous relationship. Every expansion of freedom has historically been linked to conflictual processes that challenged consolidated relations of domination; at the same time, every institutionalization of freedom has entailed more or less explicit forms of normalization, channeling, or neutralization of conflict. This tension does not represent an accidental contradiction, but rather a constitutive dynamic of modern societies. What appears specific to the contemporary conjuncture is therefore not the presence of a conflict between freedom and order, but the possibility that freedom continues to be proclaimed as a central value while conflict is systematically removed as a legitimate principle of social transformation (Crouch, 2004; Brown, 2015).

In Western democracies, individual freedom retains a hegemonic position within political and legal discourse. It functions as a criterion for legitimizing the institutional order, as a normative foundation of public policies, and as a symbolic horizon within which subjects are called upon to interpret themselves as autonomous, responsible, and capable of choice. Yet this discursive centrality coexists with structural transformations that radically call into question the emancipatory content of freedom itself. Persistently rising inequalities, the widespread precarization of living conditions, the fragmentation of collective forms of solidarity, and the normalization of modes of governance increasingly oriented toward security delineate a context in which formal freedom not only fails to guarantee processes of emancipation, but proves fully compatible with new and pervasive forms of domination (Streeck, 2014; Brown, 2018).

This gap cannot be understood as a simple inconsistency between declared principles and actual practices, nor as the contingent effect of a deviation from an original ideal that has been betrayed. Rather, it signals a profound transformation in the social and political meaning of freedom. The central thesis guiding this article is that freedom, in its currently dominant configuration, has historically consolidated as a *status*: a juridical, discursive, and symbolic status that defines subjects as formally free while remaining structurally disconnected from the material and collective processes of liberation. In this form, freedom is not simply reduced or emptied of content; it is reconfigured in such a way as to become compatible with neoliberal orderings, processes of de-democratization (Tilly, 2012), and the neutralization of social conflict (Crouch, 2011; Dardot and Laval, 2013).

To speak of freedom as a status does not mean claiming that freedom has become a fiction or an ideological illusion. On the contrary, it means recognizing that freedom functions effectively as a principle of governance. As a status, freedom attributes to subjects a form of abstract autonomy that allows structural constraints to be reinterpreted as individual choices, systemic inequalities as differences in capacity, and processes of exclusion as natural outcomes of competition. In this sense, freedom does not stand in contradiction to exploitation, precarity, or structural dependency; rather, it contributes to rendering them socially intelligible and politically manageable (Boltanski and Chiapello, 1999; Dardot and Laval, 2013).

The theoretical problem addressed in this article does not therefore concern the defense of freedom against its explicit negations, nor the nostalgic recovery of a heroic conception of liberation. It concerns the historical process through which freedom has been progressively separated from conflict and from processes of liberation, ultimately becoming a depoliticized category (de Nardis, 2017), administrable and compatible with the suspension of emancipatory conditions. The question is not whether freedom exists, but what kind of freedom is socially operative today and which forms of subjectivation it makes possible (Brown, 2015; Fraser, 2013).^1^

This tension is not merely theoretical. It is reflected in ordinary social experiences in which subjects are formally free yet increasingly constrained to adapt to precarious labor markets, conditional welfare regimes, securitized public spaces, and permanent states of emergency. In such contexts, freedom is preserved at the level of legal status and moral self-understanding, while the capacity to contest the conditions that structure everyday life is progressively weakened.

It is important to clarify from the outset that the argument developed in this article does not advance a normative hierarchy that simply opposes emancipation to freedom as such, nor does it advocate the subordination of juridical freedom to an indeterminate emancipatory horizon. Rather, the critique is directed at a historically specific configuration of freedom—namely, its stabilization as a depoliticized status—whose political effects can be analytically distinguished from freedom understood as a situated and conflictual capacity.

In this sense, emancipation is not treated as a teleological promise or as a moral imperative grounded in optimism or hope, but as a diagnostic category that allows one to assess the conditions under which freedom either expands as a socially effective practice or contracts into a merely formal attribute. The emphasis on liberation and emancipation does not imply a disregard for the protective function of legal and institutional freedoms, nor does it underestimate the historical risks associated with emancipatory projects that sever themselves from constraints, mediation, and reflexivity (Arendt, 1963; Berlin, 1969).

At the same time, approaches that prioritize freedom exclusively as a status—often in the name of preventing violence, coercion, or tyranny—tend to obscure the structural asymmetries and positional inequalities through which freedom itself is differentially produced and experienced. From this perspective, the problem is not the protection of freedom against emancipation, but the transformation of freedom into a self-legitimating principle that becomes increasingly detached from the social processes of liberation that historically rendered it meaningful and politically productive (Brown, 2015; Fraser, 2013).

To address this problem, the article explicitly adopts a *positional sociology* perspective. By this term, the article refers to a theoretical approach that takes *social position* as a key analytical node for understanding the relationship between structure, history, and subjectivity. Positions are not conceived as static locations within a social hierarchy, nor as individual attributes, but as historically produced configurations of material conditions, institutional dispositifs, symbolic grammars, and subjective dispositions that differentially structure possibilities for action, interpretation, and conflict.^2^

From this perspective, freedom, conflict, and emancipation cannot be analyzed as abstract or universally available categories, but as situated capacities that depend deeply on the positions subjects occupy within specific social arrangements. Positional sociology thus makes it possible to move beyond both purely structuralist readings, which reduce subjectivity to a mere effect of material conditions, and voluntaristic or normativist approaches, which presuppose subjects capable of freedom and agency independently of the historical and institutional conditions that make such capacities practically exercisable. In this sense, it provides a particularly suitable analytical framework for interrogating the contemporary transformation of freedom as status and the growing fragility of processes of liberation in contexts marked by precarization, depoliticization of conflict, and the normalization of war.

Political concepts are therefore not treated here as hypostatized normative entities, but as historical condensations of social relations, institutional practices, and material configurations that differentially structure possibilities for action and recognition. Within this framework, freedom is analyzed as a situated capacity, unevenly distributed according to the positions subjects occupy within specific social, economic, and institutional arrangements (Mills, 1959; Offe, 1984).

In this perspective, liberation is not conceived as a necessary outcome of history nor as a normative ideal to be abstractly opposed to the existing order. It is understood instead as a historical and conflictual process that emerges under specific conditions, starting from social positions marked by contradictions experienced as intolerable. Liberation refers to the possibility of transforming positions themselves and the social relations that produce subordination, rather than to the mere formal recognition of rights (Fraser, 2013; Somers, 2008).

A central role in the argument is finally attributed to the crisis of traditional forms of political and social mediation, which contributes to rendering freedom increasingly abstract and individualized. In contexts marked by the erosion of collective capacities for representation and by the reduction of citizenship to a formal status, freedom tends to survive as a symbolic principle precisely as the social conditions of its emancipatory translation are weakened (Crouch, 2004; Somers, 2008).

The article is structured as follows: the second section reconstructs the trajectory through which freedom has been consolidated as a juridical and discursive status, showing how its separation from conflict has facilitated its compatibility with advanced forms of domination; the third section analyzes liberation as a historical and positional process, highlighting its material and conflictual dimension; the fourth section examines the role of governmental and security dispositifs in the suspension of conditions for social emancipation; the fifth section proposes a reconceptualization of freedom in conflictual and positional terms, capable of reconnecting individual freedom and collective liberation without reverting to abstract normative models; the conclusion reflects on the implications of this perspective for a critical social theory of emancipation in the contemporary conjuncture.

## 2 Freedom as status and the neutralization of conflict

In modern political discourse, freedom is often represented as an original, almost pre-social principle that would precede relations of power and historical conflicts. This representation, however, is the outcome of a long process of abstraction that has progressively separated freedom from the social conditions of its production. From the standpoint of social theory, freedom never emerges in an empty space, nor can it be understood as the mere absence of constraints. It always takes shape within historically determined relations of force and is inseparable from the conflicts that delimit its content, subjects, and limits (Polanyi, 1944; Macpherson, 1962). Every extension of freedom has historically been the product of struggles that challenged consolidated arrangements; at the same time, every institutionalization of freedom has entailed a redefinition of order aimed at stabilizing certain conflicts while neutralizing others.

This dynamic is constitutive of political modernity. Freedom does not assert itself against order, but through a transformation of order itself, selectively incorporating certain conflictual demands while excluding others. What is recognized as legitimate freedom is always the result of a historical compromise among social forces, rather than the expression of a neutral and universal principle. In this sense, freedom cannot be understood as an abstract good, but as a historically situated social form whose meaning shifts in relation to transformations in economic structures, political institutions, and configurations of power (Dumont, 1986; Skinner, 1998).

It is precisely this historicity that tends to be obscured by the neoliberal conception of freedom, which presents itself as a minimal and universalizable definition. Yet even a brief genealogical inquiry is sufficient to call this claim into question. Even within the Western context, the idea of freedom does not originally emerge as a principle of separated individual autonomy, but is instead closely linked to dimensions of belonging, status, and collective recognition. Similarly, in many non-Western traditions, freedom is not conceived as the absence of interference, but as a relational condition, referring to a recognized status within a social order rather than to an individual sphere detached from social bonds. This genealogical indication is not intended to propose alternative models, but to denaturalize modern negative freedom by showing that it constitutes a specific historical configuration rather than an anthropologically universal form (Patterson, 1991; Mehta, 1999).^3^

Western negative freedom, as it consolidates within liberal modernity, thus represents a profound and non-obvious transformation of the concept of freedom. It presupposes a sharp separation between individual and community, a neutralization of social conflict, and a conception of freedom as a space of non-interference guaranteed by the legal order. This reconfiguration does not eliminate the historical dimension of freedom, but reorganizes it in such a way as to render it compatible with advanced forms of governance and domination. It is in this passage that freedom progressively comes to assume the form of a status (Berlin, 1969; Hayek, 1960).

Over the course of the twentieth century, and with particular intensity in the neoliberal phase, freedom loses its character as a historical practice linked to the contestation of relations of domination and becomes stabilized as a formal attribute of the subject. As a status, freedom becomes a juridical and discursive condition attributed to individuals as such, independently of the material and relational conditions that make its effective exercise possible or impossible. This transformation does not imply a quantitative reduction of freedom, but a qualitative restructuring. Freedom as status is radically individualized and refers to an abstract subject, formally equal to others, separated from the social relations that differentially structure access to resources, security, and recognition (Macpherson, 1962; Dumont, 1986).

At the same time, freedom as status entails a systematic depoliticization of conflict (de Nardis, 2020). If freedom is conceived as an individual space of choice or non-interference, conflict can only appear as an external and disruptive element to be contained or neutralized. Social contradictions are recoded as technical problems, administrative dysfunctions, or moral issues, while collective struggles lose their status as legitimate practices of transformation. In this framework, conflict is no longer recognized as a constitutive dimension of freedom, but as a threat to the order that freedom is supposed to guarantee (Berlin, 1969; Pettit, 1997).

From a sociological perspective, this transformation becomes visible in situations in which individuals are expected to demonstrate autonomy precisely by accepting constraints they did not choose: precarious employment presented as opportunity, reduced social protection framed as incentive, or exposure to risk reinterpreted as personal responsibility. In these contexts, freedom operates less as a capacity to shape social conditions than as a requirement to adapt to them.

This dynamic can be observed with particular clarity across different historical and political domains, in which the tension between freedom and liberation takes specific but structurally analogous forms. In the case of feminism, for example, movement genealogies show how the question of liberation was originally formulated in explicitly structural and collective terms. Over time, and especially in its liberal–mainstream variants, this horizon has been progressively translated into the language of individual freedom, understood as choice, self-realization, and success within the market. In this shift, liberation tends to survive as a rhetorical reference, while freedom takes the form of individual performance, compatible with the persistence of the very relations of domination that feminist movements initially sought to transform (Brown, 1995).

A similar tension runs through postcolonial processes, in which the acquisition of juridical and formal freedom—in the form of independence, sovereignty, and international recognition—has not coincided with genuine liberation from economic dependencies, geopolitical constraints, and global hierarchies. Political independence does not automatically translate into social emancipation and can coexist with new forms of subordination, often mediated by national elites. Here again, freedom operates as a stabilized juridical form, while liberation remains an unfinished and conflictual process (Mehta, 1999; Patterson, 1991).

It is within the neoliberal context, however, that this separation acquires a systemic function. Neoliberalism represents a historical regime that multiplies the vocabulary of freedom—choice, flexibility, autonomy, responsibility—while systematically neutralizing any project of liberation. Structural conflicts are individualized, inequalities are translated into differences in behavior and lifestyles, and the capacity for adaptation (resilience) is elevated to a criterion of freedom. Within this framework, liberation—understood as the questioning of social relations through resistance—tends to be delegitimized as ideological nostalgia, political irrationality, or a threat to order. Neoliberal freedom thus functions not as a premise, but as a preventive dispositif against any possible process of liberation (Harvey, 2005; Foucault, 2008).

This reconfiguration is further reinforced by a process of moralization that plays a central role in contemporary governance (de Nardis, 2026b). Freedom, understood as individual autonomy, is accompanied by an imperative of responsibility that transforms structural constraints into personal choices and systemic inequalities into differences of merit. The capacity to adapt to precarious conditions, to assume risks, and to compete in unstable environments is presented as an expression of freedom, while failure or vulnerability tend to be interpreted as individual deficits. In this sense, freedom does not oppose domination, but contributes to rendering it socially acceptable by translating relations of power into differences of behavior (Brown, 1995; Harvey, 2005).

The outcome of these processes is a paradoxical yet stable configuration: a formally extended freedom that coexists with the systematic erosion of the conditions of liberation. Freedom as status can be guaranteed even in the absence of material security, social protection, and collective capacities for action. It does not require the elimination of relations of domination, but only their translation into forms compatible with abstract individual autonomy. In this sense, the separation between freedom and liberation does not represent an anomaly or an unresolved contradiction, but the coherent outcome of a historical transformation that has rendered freedom administrable and politically neutral.

Understanding freedom as status therefore means recognizing that it is not simply threatened or emptied of content, but that it functions as a dispositif of governance. Precisely because it is depoliticized, freedom can survive symbolically even as the possibilities for social transformation are drastically reduced. It continues to operate as a principle of legitimation of order while conflict is marginalized and liberation rendered impracticable. It is from this diagnosis that it becomes possible to interrogate liberation not as a lost normative ideal, but as a historical and positional process whose fragility constitutes one of the most significant indicators of the contemporary conjuncture.

## 3 Liberation as a historical and positional process

If freedom, in its dominant configuration, tends to stabilize as a juridical and discursive status, *liberation* refers to a radically different dimension of social and political experience. Unlike freedom, liberation cannot be attributed, guaranteed, or administered: it is not a condition one possesses, but a process one undergoes. To speak of liberation therefore means shifting the analysis from the plane of stabilized forms of order to that of historical transformations that call into question their material, symbolic, and relational foundations. In this sense, liberation does not constitute a complement to freedom, but rather its critical reversal: that which renders visible the historical and contingent character of what appears natural or necessary [Marx, 1975 (1844); Luxemburg, 2008 (1900)].

Within the tradition of critical social theory, liberation has never been conceived as a final state or as a destiny inscribed in the course of history. It emerges instead in moments of rupture, when structural contradictions are experienced as intolerable and cease to be interpreted as individual misfortunes or temporary dysfunctions. Liberation does not coincide with the expansion of rights nor with the formal recognition of subjects; it entails the contestation of the social relations that render those rights abstract, selective, or inaccessible. For this reason, liberation is inseparable from conflict—not as a contingent episode of disorder, but as the historical modality through which the social order is stripped of its claim to necessity (Thompson, 1963; Tilly, 2004).

From a positional sociology perspective, liberation can be understood only in relation to the material and symbolic conditions within which subjects are situated. Social positions do not merely constitute static locations within a hierarchy, but historically produced configurations of resources, constraints, expectations, and vulnerabilities that differentially structure possibilities for action and interpretation. Liberation thus does not arise from an abstract will to emancipation, but from the encounter between material conditions of subordination and collective processes of interpretation that render those conditions intelligible as the outcomes of transformable social relations (Bourdieu, 1998, 2000).

In this sense, liberation is never universally available. It is always positional, situated, and partial. What is at stake here is not simply unequal access to resources, but unequal exposure to the costs of conflict itself. For subjects occupying protected positions, dissent may remain a reversible practice; for those in exposed positions, the same gesture can entail immediate material, social, or legal sanctions. Positional sociology makes this asymmetry analytically visible. Some subjects possess material, organizational, and symbolic resources that make it possible to translate social suffering into conflict; others, located in more exposed positions, encounter obstacles that tend to convert suffering into adaptation, resignation, or guilt. Positional sociology allows us to grasp this asymmetry with precision: the very possibility of thinking liberation depends on the positions subjects occupy and on the protections—or lack thereof—that accompany them (Bourdieu, 2000).

This positional dimension helps clarify the structural distance between freedom and liberation. While freedom as status can be formally extended to all subjects regardless of their material conditions, liberation requires a transformation of positions themselves. It entails a reorganization of social relations, institutions, and forms of recognition that produce subordination and dependence. In this sense, liberation cannot be fully translated into the language of law without losing its conflictual content. Any attempt to institutionalize it tends to stabilize its outcomes, separating them from the historical process that made them possible and neutralizing the transformative charge that animated them (Fraser and Honneth, 2003; Fraser, 2009).

It is precisely this feature that renders liberation a theoretically uncomfortable category in the contemporary conjuncture. In a historical phase marked by the depoliticization of conflict, the fragmentation of subjectivities, and the individualization of risk, liberation appears as an excessive concept, difficult to reconcile with an order that privileges the administrative management of social problems. Freedom, as status, can be administered; liberation, as process, cannot. It exceeds existing institutional frameworks and calls into question the material foundations of the social order, making visible the power relations that abstract freedom tends to obscure (Touraine, 1981; Burawoy, 2005).

The progressive marginalization of the lexicon of liberation is therefore not a sign of its historical obsolescence, but an indicator of a transformation in the conditions of possibility for collective action. The precarization of labor, the erosion of intermediary bodies, generalized competition, and the normalization of insecurity reduce subjects' capacity to recognize themselves as bearers of shared interests. In the absence of such recognition, conflict tends to fragment or to be reabsorbed into forms compatible with the reproduction of the existing order. Deprived of its material and organizational bases, liberation is thus replaced by narratives of individual autonomy that leave the structure of inequalities intact (Wright, 2010).

Understanding liberation as a historical and positional process makes it possible to avoid both teleological conceptions of emancipation and normativist reductions of freedom. Liberation is not an ideal to be opposed abstractly to reality, but a fragile historical possibility, always exposed to the risk of regression, capture, or neutralization. It does not secure freedom once and for all, but constitutes its dynamic condition of renewal. In the absence of processes of liberation, freedom tends to harden into the form of status; in the absence of freedom, liberation risks dissolving into a mere negation of the existing order (Luxemburg, 2008 (1900); Wright, 2010).

It is from this unresolved tension that it becomes possible to interrogate the role of war and securitarian dispositifs in the contemporary conjuncture. If liberation presupposes material and collective conditions of subjectivation, war—understood not as an exceptional event but as a structural dispositif—intervenes precisely upon those conditions, redefining them in restrictive terms. Analyzing this nexus makes it possible to understand how abstract freedom can survive formally even when processes of liberation are systematically suspended.

## 4 War, security, and states of exception

If freedom as status can survive its separation from social conflict, this is because the contemporary order disposes of dispositifs capable of preserving its formal architecture while progressively hollowing out its emancipatory content. Among these dispositifs, war occupies a central position—not only as a destructive or violent phenomenon, but as an ordering principle that reorganizes political priorities, redefines social hierarchies, and intervenes directly in the conditions of possibility of collective subjectivation. From a social-theoretical perspective, war cannot be treated as an exceptional event that temporarily interrupts democratic normality; rather, it must be understood as a structural dimension of contemporary governance, capable of profoundly reshaping the very meaning of freedom (Foucault, 2003; Shaw, 2005; de Nardis, 2025).

Contemporary wars—direct, indirect, proxy, hybrid, and technologically mediated—do not merely produce geopolitical effects. They operate as dispositifs of social restructuring, intervening in the distribution of resources, the legitimation of authority, and the definition of collective urgencies. War introduces a specific political temporality, dominated by emergency, necessity, and the anticipation of threat. Within this compressed temporality, the future no longer appears as a space of transformation, but as a horizon of risk to be managed. This temporal reconfiguration has decisive consequences for emancipatory processes: it makes the construction of long-term collective projects more difficult and reinforces a rationality of adaptation that privileges individual survival over social transformation (Virilio, 2002; Duffield, 2007).

At this point, a crucial theoretical distinction is required in order to avoid any transhistorical or reductive understanding of the relationship between war, conflict, and emancipation. Historically, war has not been a homogeneous phenomenon, nor has it played a univocal role with respect to processes of liberation and democratization. Different forms of war have produced profoundly different political and social effects, which cannot be collapsed into a single analytical category (Tilly, 1992; Mann, 1988).

In particular, at least three analytically distinct configurations must be distinguished. First, wars of liberation and resistance—typically emerging from conditions of colonial domination, occupation, or radical political oppression—have often functioned as moments of collective politicization and as catalysts for emancipatory aspirations. Their historical ambivalence is undeniable, yet they cannot be reduced to a mere negation of freedom, insofar as they were embedded in struggles aimed at transforming asymmetrical relations of domination.

Second, the experience of total war in the twentieth century—most notably during the First World War—has been widely interpreted as having indirectly contributed to processes of political inclusion and democratization, through the expansion of mass mobilization, social rights, and state capacities. As classical analyses have shown, the extension of citizenship, welfare arrangements, and political participation was in part intertwined with the extraordinary demands imposed by total war on modern societies (Finer, 1975; Tilly, 1992; Poggi, 1978; Kaldor, 1999).

The argument developed in this article, however, concerns neither wars of liberation nor the historically specific dynamics of total war. It focuses instead on the distinctive configuration of contemporary wars and security regimes, which operate as technologies of governance rather than as moments of collective politicization. In this third configuration—characteristic of late-modern neoliberal societies—war no longer functions as an external rupture capable of reconfiguring social contracts, but as a permanent condition that reorganizes social life around risk management, emergency, and securitization (Duffield, 2007; Shaw, 2005; Foucault, 2003).

It is in this sense that war can be analytically understood as a negation of conflict rather than as its radicalization. By externalizing antagonism, moralizing dissent, and subordinating internal social contradictions to the imperative of security, contemporary forms of war contribute to the depoliticization of social conflict and to the erosion of the collective conditions required for processes of liberation and emancipation (Agamben, 2005; Bigo, 2002; Bauman, 2006).

The central theoretical point is that war does not suspend freedom as such, but rather reorganizes its social conditions of exercise. Freedom as a juridical status can remain formally intact even in contexts of war or permanent threat, precisely because what is suspended are not declared rights, but the material and collective conditions that make their translation into emancipatory practices of subjectivation possible. In this sense, war does not represent the antithesis of liberal freedom, but one of the conditions of its full realization as a governing principle. Freedom is recoded as individual protection, responsible conformity, and willingness to accept constraints presented as inevitable (Agamben, 2005; Foucault, 2003).

From a positional sociology perspective, war acts first and foremost on the distribution of vulnerabilities. It does not affect subjects uniformly, but reorganizes social positions through an intensification of existing asymmetries. Some subjects, located in relatively protected positions, experience war primarily as narrative and symbolic horizon; others, situated in more exposed positions, experience its direct material effects, such as rising living costs, the erosion of social protection, labor precarization, and the militarization of public space. Positions thus shape not only access to resources, but also the very ways in which war is perceived, interpreted, and incorporated into subjective dispositions (Bauman, 2006; Butler, 2004).

Within this framework, securitarian dispositifs assume a decisive role. Security, prevention, risk, and emergency have become central categories of everyday governance, through which war is institutionalized in social life even in the absence of direct armed conflict. Security does not merely protect; it classifies, selects, and hierarchizes, differentially assigning degrees of trustworthiness and suspicion. In this way, war produces a normalization of control and an internalization of threat, while freedom is reinterpreted as the conscious acceptance of “necessary” limitations and as willingness to conform to procedures presented as rational and unavoidable (Bigo, 2002; Duffield, 2007).

Under such conditions, war is no longer encountered primarily as an exceptional event, but as a diffuse background shaping everyday expectations: acceptance of restrictions in the name of security, moralization of dissent, and the portrayal of social conflict as irresponsible in times of crisis. These processes affect social positions unevenly, reinforcing the distance between those who can afford dissent and those for whom dissent becomes prohibitively costly.

War also intervenes deeply in processes of subjectivation. It produces a specific affective regime centered on fear, alarm, resentment, and the desire for order. These dispositions cannot be reduced to individual psychological states; they must be understood as socially produced effects, emerging from the interplay between material precariousness, symbolic insecurity, and institutional narratives that define the present as a condition of permanent crisis. In such a context, the capacity to interpret one's conditions as the product of transformable social relations tends to weaken, while the propensity to read vulnerability as individual fate or as the inevitable consequence of external threats is reinforced (Butler, 2004; Bauman, 2006).

It is in this sense that war operates as a dispositif for the suspension of emancipatory conditions. Liberation, understood as a collective process of transforming social positions, presupposes minimal conditions of stability, mutual recognition, and organizational capacity. War acts in the opposite direction: it fragments subjectivities, polarizes attachments, redirects conflict outward, and delegitimizes forms of internal contestation. Even in the absence of direct repression, social conflict is rendered untimely, irresponsible, or morally unacceptable, while structural contradictions are subordinated to the priority of security and cohesion (Hardt and Negri, 2004; Mbembe, 2003).

In this process, the distinction between autocracies and formal democracies tends to lose part of its explanatory power. Without denying relevant institutional differences, it is necessary to recognize that war produces functional convergences: the centralization of decision-making, the strengthening of the executive, the contraction of deliberative spaces, the moralization of political loyalty, and the delegitimation of dissent. In formal democracies, these processes can unfold without explicit constitutional ruptures precisely because freedom as status remains intact and continues to legitimize the order. What is compromised is not declared freedom, but the possibility of exercising it as a conflictual practice oriented toward transformation (Agamben, 2005; Foucault, 2003).

From a positional perspective, war thus contributes to redefining the map of emancipatory possibilities through a dual operation: on the one hand, it intensifies inequalities and renders collective action more fragile; on the other, it produces a political imaginary in which liberation appears impracticable or even dangerous, while freedom is reduced to individual protection and responsible adaptation. In this sense, war is not merely an external context that limits freedom, but a dispositif that restructures its social meaning and reinforces its governing function (Duffield, 2007; Mbembe, 2003).

If the contemporary conjuncture is characterized by the coexistence of formal freedom and emancipatory suspension, this is because freedom and war are not antithetical concepts in the way neoliberal discourse tends to suggest. War can coexist perfectly with freedom understood as status, and can even reinforce it symbolically by transforming it into an identity marker opposed to an external enemy. What war systematically erodes are the material and collective conditions of liberation—that is, the possibility of transforming social conflict into an emancipatory force.

It is from this diagnosis that it becomes necessary to interrogate freedom itself once again. If freedom as status is compatible with neoliberal order and permanent war, it becomes imperative to develop a notion of freedom capable of reintegrating conflict within it and of recomposing the nexus between individual freedom and collective liberation. This theoretical passage constitutes the pivotal point of the argument and prepares the reconceptualization proposed in the following section.

## 5 For a conflictual and positional conception of freedom

If the previous analyses have shown how freedom can be separated from conflict and stabilized as a status compatible with neoliberal order and permanent war, the theoretical problem that now emerges is not that of abandoning freedom as a critical category, but of rescuing it from its depoliticized definition. The task of a social theory of freedom does not consist in opposing existing freedom with an alternative normative ideal, but in reconstructing the historical and social conditions under which freedom can once again operate as a transformative practice. From this perspective, the question is not whether freedom is still possible, but which form of freedom is socially practicable today, and under what conditions (Berlin, 1969; Sen, 1999).

From the standpoint of critical sociology, freedom cannot be conceived either as a sphere protected from interference or as an intrinsic property of the subject. Such conceptions presuppose a social world already pacified, in which relations of force have been neutralized or rendered invisible. Yet in societies structurally traversed by inequalities, power asymmetries, and differentially vulnerable positions, freedom can only be understood as a situated capacity. It does not precede social relations, but emerges within them; it is not exercised outside constraints, but through the ways in which those constraints can be questioned, renegotiated, or transformed (Lukes, 2005; Bourdieu, 2000).

Reconceptualizing freedom in conflictual terms first requires recognizing that it is not primarily expressed in the choice among given options, but in the capacity to intervene in the field of options itself. Freedom does not coincide with adaptation to an existing order, but with the ability to make that order an object of conflict, to strip social conditions of their claim to necessity, and to open spaces for transformation. This conception deliberately resists both liberal accounts that reduce freedom to individual choice and romantic accounts that equate conflict with emancipation *per se*. This also implies a distance from approaches that treat emancipation as a normative horizon to be privileged over juridical freedom in the name of historical necessity or moral hope.

The argument developed here does not endorse a teleological primacy of emancipation over freedom, but insists on their fragile and conflictual co-dependence under conditions that include protection, mediation, and institutional constraint. What is at stake is not the celebration of conflict, but the social conditions under which conflict can become a sustainable practice rather than a source of individual exhaustion or punishment. In this sense, freedom is not simply a matter of individual decision, but of power to affect the structures that define what can be said, thought, and practiced (Lukes, 2005; Dean, 2010).

This capacity, however, is not universally available. It depends on the positions subjects occupy, on the material and symbolic resources at their disposal, and on the institutional protections that either shield them or expose them to risk. Freedom, understood as a conflictual practice, is therefore intrinsically unequal: some subjects can exercise it without immediate costs, while for others it entails high risks of marginalization, repression, or loss of material security. Here the limits of conceptions of freedom grounded exclusively in individual capabilities become evident, insofar as such capabilities are not traced back to the social conditions that make their effective exercise possible (Sen, 1999; Bourdieu, 2000).

It is at this point that the perspective of positional sociology reveals its full theoretical relevance. If freedom is a positional capacity, it cannot be conceived exclusively at the level of the isolated individual. Individual freedom is not an original given, but the outcome of collective configurations that make action possible without immediately translating it into unsustainable vulnerability. In this sense, individual freedom and collective liberation are not alternative terms, but intertwined dimensions of the same historical process. The freedom of the individual presupposes the existence of collective conditions that sustain it; collective liberation, in turn, is nourished by individual practices of dissent that destabilize the existing order (Rancière, 1999; Mouffe, 2005).

A conflictual conception of freedom thus makes it possible to move beyond the classic opposition between negative and positive liberty without resolving it into a normative synthesis. The issue is not whether freedom consists in the absence of interference or in participation in a collective project, but how and when subjects are able to intervene upon the constraints that define them. Freedom is neither pure individual autonomy nor harmonious communal integration; it is an unstable practice located in the space of tension between subjectivity and structure, lived experience and institutional transformation. It manifests itself in moments of disagreement, rupture of consensus, and rearticulation of the political (Rancière, 1999; Mouffe, 2005).

From this perspective, conflict does not represent a threat to freedom, but its condition of possibility. Without conflict, freedom tends to harden into the form of status; without freedom, conflict risks collapsing into a mere negation devoid of emancipatory outcomes. The point is not to celebrate conflict as such, but to recognize its constitutive function in processes of subjectivation and social critique. It is through conflict that inequalities become visible, that subjects cease to adapt to heteronomous forms of life, and begin to call them into question (Jaeggi, 2018).

Conflictual freedom is, by definition, incomplete and reversible. It cannot be guaranteed once and for all, nor fully translated into juridical norms without losing its transformative force. Every institutionalization of freedom entails a risk of normalization: what emerges as a practice of rupture can be absorbed, regulated, and rendered compatible with the existing order. This risk is particularly evident in political articulation processes that transform emancipatory demands into stabilized political identities, sometimes neutralizing their original conflictual charge (Laclau, 2005).

Yet this risk does not justify abandoning freedom as a critical category. On the contrary, it calls for theoretical vigilance with respect to the social conditions that allow freedom to remain a living practice rather than a mere formal attribute. In a context marked by widespread precarization, fragmentation of subjectivities, and the normalization of war, freedom tends to be reduced to capacities for adaptation, resilience, and individual risk management. Reintroducing conflict at the core of freedom instead means challenging this reduction and reopening the space for forms of subjectivation oriented toward the transformation of conditions of existence (Dean, 2010; Jaeggi, 2018).

From this perspective, freedom can be defined as the always situated and always fragile capacity to transform one's own position without being immediately punished or repressed for doing so. This formulation makes it possible to hold together freedom and protection, conflict and solidarity, dissent and institutions. It shifts attention from freedom as an individual property to freedom as a socially sustained process, rendering visible the structural nexus between freedom and liberation. Only where collective conditions exist that make conflict practicable without annihilating those who exercise it can freedom once again function as an emancipatory principle.

It is on the basis of this conflictual and positional conception of freedom that emancipation can be reintroduced not as an abstract ideal, but as a concrete historical tension. Emancipation does not designate a point of arrival, but the unstable movement through which societies call their forms of domination into question. Understanding the conditions of possibility of this movement, and the reasons for its current fragility, constitutes the concluding step of the argument.

## 6 Emancipation as a theoretical problem

The theoretical trajectory developed in this article begins from an observation that, while recurrent in public discourse, is rarely addressed in its deeper sociological implications: the coexistence of a persistent emphasis on individual freedom with a progressive weakening of the material, collective, and symbolic conditions of emancipation. Far from constituting a simple inconsistency between proclaimed principles and effective practices, this coexistence points to a structural transformation in the very meaning of freedom and in its relationship to conflict and liberation—a transformation that directly concerns contemporary modes of governing complex societies.

The analysis has shown how freedom, in its currently hegemonic configuration, has historically consolidated as a status. As a status, freedom is neither denied nor curtailed, but stabilized as a formal attribute of the subject, separated from the social processes that make its effective exercise possible. This separation does not represent an accidental distortion or a mere loss of content, but a reconfiguration coherent with the governing requirements of advanced neoliberal societies. Freedom as status can coexist with growing structural inequalities, widespread precariousness, and institutionalized violence precisely because it does not presuppose the transformation of the social relations that produce these conditions, but only their differential management within an order that tends to present itself as lacking structural alternatives (Fraser, 2022).

Within this framework, liberation emerges not as the missing complement of freedom, but as what abstract freedom structurally tends to remove. As a historical and conflictual process, liberation renders visible the material and relational conditions of subjectivation, depriving the social order of its claim to necessity and neutrality. For this very reason, it cannot be easily translated into the language of law or administered through institutional dispositifs without losing its transformative charge. The marginalization of the lexicon of liberation therefore does not signal its theoretical obsolescence, but rather the growing difficulty of sustaining collective processes of transformation in a context marked by the depoliticization of conflict and the naturalization of forms of domination—processes that critical social theory is called upon to render problematic once again (Jaeggi and Wesche, 2009).

The analysis of war and securitarian dispositifs further clarifies this dynamic. War, understood as an endemic and normalized dimension of contemporary governance, does not suspend freedom in a formal sense, but intervenes in the social conditions of its practicability. It reorganizes freedom as individual protection, responsible adaptation, and acceptance of constraints presented as inevitable, while rendering increasingly fragile the possibility of translating structural contradictions into organized social conflict. In this sense, war does not represent the opposite of liberal freedom, but one of the dispositifs through which it is fully integrated as a governing principle, reinforcing an order that legitimizes itself precisely through the permanent management of crisis.

The adoption of a positional sociology perspective has made it possible to avoid both a purely normative reading of freedom and an abstract conception of emancipation. Conceived as situated capacities, freedom and liberation appear inseparable from the social positions that differentially structure access to resources, protection, and recognition. Freedom is not a universal property of the subject, but a differentially distributed capacity; liberation is not a guaranteed outcome, but a fragile historical possibility, always exposed to the risks of regression, capture, or neutralization. In this sense, the weakening of emancipatory capacities is inseparable from a broader impoverishment of social relations and of the possibilities of responding to the world—an impoverishment that manifests itself as a loss of resonance between subjects, institutions, and social structures (Rosa, 2019).

From this perspective, emancipation cannot be conceived either as a teleological endpoint or as a normative ideal abstractly opposed to the existing order. The fragility of emancipation today is thus not an abstract theoretical condition, but a lived social reality, experienced through insecurity, moralized responsibility, and the narrowing of spaces in which collective dissent can be articulated. Rather, it takes the form of a permanent tension traversing modern societies—a tension that does not resolve itself into pacified syntheses, but keeps open the fracture between what is and what could be otherwise. Emancipation does not coincide with the expansion of rights nor with the mere presence of conflict, but with the historically situated capacity to articulate conflict, protection, and solidarity in ways that render a form of freedom practicable that is not reduced to status. In this sense, emancipation retains an eminently negative dimension, understood as a mode of critique that refuses the identification of the existing with the rational and insists on the non-identity between social order and human possibilities (Adorno, 1966).

The theoretical contribution of this article therefore does not lie in offering a new normative definition of freedom or liberation, but in proposing an analytical framework capable of critically recomposing the nexus between freedom, conflict, and emancipation from the standpoint of the material and positional conditions of subjectivation. In a conjuncture marked by widespread precarization, the normalization of war, social acceleration, and the erosion of collective capacities for action and response, this perspective makes it possible to understand why freedom can survive as a symbolic and juridical principle precisely when emancipation becomes increasingly fragile at the social level (Fraser, 2022; Rosa, 2019).

If emancipation today appears as an uncertain and contested horizon, this is not due to its theoretical irrelevance, but to the transformation of the historical conditions that once made its emergence possible. Bringing the nexus between freedom, conflict, and position back to the center of analysis ultimately means restoring emancipation to its status as a crucial theoretical problem for contemporary critical sociology—not as a nostalgic promise or abstract utopia, but as a field of real tensions, persistent negativities, and unreconciled possibilities within which the concrete forms of subjectivation, transformation, and conflict in present-day societies are at stake (Jaeggi and Wesche, 2009).

## Data Availability

The raw data supporting the conclusions of this article will be made available by the authors, without undue reservation.

## Footnotes

1 Throughout the article, the terms *freedom, liberation*, and *emancipation* are used in a deliberately differentiated manner. *Freedom* refers to a juridical and discursive status, historically associated with liberal and neoliberal forms of governance, and primarily understood as individual autonomy or non-interference. *Liberation*, by contrast, designates a historical and conflictual process through which existing social relations are contested and transformed. It refers to collective struggles, material conditions, and positional dynamics that cannot be fully institutionalized or administratively guaranteed. *Emancipation* is employed to indicate the broader critical horizon within which the tension between freedom and liberation is theorized. Rather than naming a final state or normative ideal, emancipation refers to the fragile and historically situated possibility of transforming social positions and relations of domination. This terminological distinction is intended not as a purely semantic exercise, but as an analytical strategy to avoid conflating juridical freedom with non-teleological and historically contingent processes of social transformation.

2 The term *positional sociology* refers to a materialist-oriented theoretical framework that takes *position* as a central analytical category for reconnecting macro-structural transformations, historical processes, and forms of subjectivity. The approach builds on a critical re-reading of historical materialism, integrated with contributions from field theory, subaltern studies, and post-structuralism, with the aim of overcoming both structuralist reductions and voluntaristic conceptions of subjectivity. From this perspective, position does not coincide with a simple objective location within a social structure, nor with a subjective standpoint, but designates a historically produced configuration of material conditions, institutional dispositifs, symbolic grammars, and practical dispositions. Positional sociology thus conceives subjectivity as the outcome of unstable positional mediations through which individuals and groups experience the contradictions of contemporary capitalism. In this sense, position operates as a bridging category between analytical levels that are often treated separately in social theory, allowing for an articulation of structure and agency without presupposing abstract subjects or mechanical forms of determinism. The approach was initially developed within a network of scholars active in the Italian context (the *Rete di Sociologia di Posizione*), but it is explicitly situated in dialogue with international debates on processes of neoliberalization, social decomposition, and the micro–macro relationship in contemporary social sciences (de Nardis and Simone, 2022; de Nardis et al., 2023; de Nardis, 2026a,b).

3 The reference to non-Western conceptions of freedom serves here an exclusively critical and genealogical function. It does not aim to provide a systematic reconstruction of different linguistic and cultural traditions, nor to propose alternative models to be adopted normatively. Rather, it is intended to underscore that modern negative freedom cannot be treated as an anthropologically minimal or transhistorical definition. Genealogical comparison instead makes it possible to show that the association between freedom and individual autonomy is the outcome of a specific historical trajectory, tied to particular political, social, and economic configurations. In this sense, the reference to extra-Western semantic fields does not introduce a relativistic argument, but rather reinforces the claim that the naturalization of modern freedom tends to obscure the historically situated and conflictual character of emancipatory processes. For an etymological and institutional reconstruction of the semantics of freedom, see Benveniste (1969) and the historical-sociological comparison proposed by Patterson (1991).
